# Medical Professionals’ Responses to a Patient Safety Incident in Healthcare

**DOI:** 10.3389/ijph.2024.1607273

**Published:** 2024-07-26

**Authors:** Lucia Kupkovicova, Ivana Skoumalova, Andrea Madarasova Geckova, Zuzana Dankulincova Veselska

**Affiliations:** ^1^Institute of Applied Psychology, Faculty of Social and Economic Sciences, Comenius University, Bratislava, Slovakia; ^2^Department of Health Psychology and Research Methodology, Faculty of Medicine, University of Pavol Jozef Šafárik, Košice, Slovakia

**Keywords:** patient safety incidents, medical professionals, healthcare, psychological safety, patient safety

## Abstract

**Objectives:** Patient safety incidents (PSIs) are common in healthcare. Open communication facilitated by psychological safety in healthcare could contribute to the prevention of PSIs and enhance patient safety. The aim of the study was to explore medical professionals’ responses to a PSI in relation to psychological safety in Slovak healthcare.

**Methods:** Sixteen individual semi-structured interviews with Slovak medical professionals were performed. Obtained qualitative data were transcribed verbatim and analysed using the conventional content analysis method and the consensual qualitative research method.

**Results:** We identified eight responses to a PSI from medical professionals themselves as well as their colleagues, many of which were active and with regard to ensuring patient safety (e.g., notification), but some of them were passive and ultimately threatening patients’ safety (e.g., silence). Five superiors’ responses to the PSI were identified, both positive (e.g., supportive) and negative (e.g., exaggerated, sharp).

**Conclusion:** Medical professionals’ responses to a PSI are diverse, indicating a potential for enhancing psychological safety in healthcare.

## Introduction

Providing healthcare entails significant risks of patient safety incidents (PSIs), which are defined as events that “could have resulted, or did result, in unnecessary harm to a patient” [1, p. 15]. WHO (2009) differentiates four types of PSIs that are caused by errors or violations: reportable circumstances, near misses, no-harm incidents and adverse events [1]. Although the number of PSIs in the United States appears to have declined significantly over the past decade [2], it does not seem to be a global trend [3, 4]. Recent systematic reviews and meta-analyses show that PSIs still represent a significant threat to patient safety worldwide: approximately one in twenty patients is exposed to a preventable adverse event [5] and one in thirty patients has experience with a preventable medication adverse event [6]. In Slovakia, the recent study conducted during COVID-19 found that one-third of medical professionals had witnessed or heard of PSI in the past year [7]. Therefore, the global goal is to strengthen patient safety in healthcare and ensure a reduction in preventable PSIs [8].

Each healthcare provider in Slovakia have to implement a quality management system, which includes regular clinical audit [9]. Clinical audit includes the verification of compliance with the internal patient safety assessment system and the fulfilment of the minimum requirements for the internal patient safety assessment system [9]. The minimum requirements for the internal patient safety assessment system are set out in the Decree of the Ministry of Health of the Slovak Republic no. 444/2019, which is effective as of 1 January 2020 [10].

Besides that, Healthcare Surveillance Authority issued the methodological guideline in 2014, which differentiate two PSI reporting systems in institutional healthcare facilities [11]. As part of the mandatory reporting system, medical professionals are required to report the occurrence of a serious adverse event and fill out a standard protocol about it [11]. The voluntary reporting system is for medical professionals to voluntarily and informally report errors and near-misses in order to learn from them [11]. However, recent studies indicate that reporting of PSIs in Slovakia is insufficient [7, 12]. In addition, root-cause analysis of adverse events does not seem to be a standard practice in Slovakia [7].

Patient safety could be endorsed by medical professionals’ open communication about patient safety concerns [13] and about PSIs [14]. Additionally, open and transparent communication serves as an organisational factor that supports PSI reporting in healthcare [15]. The reporting of PSIs further allows for root cause analysis to take place, enabling medical professionals to learn from incorrect practices, to find preventive solutions and to implement them in practice [8]—all of which contribute to the safety of patients.

In an organisational context, voice behaviour is facilitated by psychological safety [16] that is defined as a work climate in which it is safe to express opinions or concerns without subsequently having to face negative reactions and consequences from superiors or colleagues [17, 18]. Specifically in healthcare, the perceived safety of speaking up is an important factor involved in medical professionals’ decision to speak up [19]. Psychological safety in a clinical workplace is associated with open and respectful interpersonal communication and medical professionals’ ability to draw the attention of their colleagues or superiors to PSIs [13]. According to O’Donovan and McAuliffe’s (2020) systematic review, psychological safety in healthcare is facilitated mostly by support from organisation, leaders and peers and by the emphasis on patient safety [20].

An unproductive form of speaking up also occurs in an organisational context, which could negatively affect the psychological safety and the ability to speak up [21]. Irrelevant comments, outbursts of anger, insults, or even threats can have harmful effects not only on those who are the target of communication, but also on other employees who witness the situation or only hear about it [21]. The research study showed that medical professionals working in clinical workplaces with low psychological safety had experiences with inappropriate and negative tone of communication from superiors, and felt less safe to speak up [13].

In this sense, medical professionals’ immediate responses in the aftermath of a PSI might reflect a level of psychological safety in their workplace. However, the previous research studies focused either on the experiences of medical professionals after a PSI [22], or on psychological safety in speaking up about patient safety concerns [13], whereas we perceived a lack of research studies that would link these two topics. Therefore, the aim of the present study was to explore Slovak medical professionals’ responses to a PSI in the context of psychological safety. The study was focused on medical professionals’ own responses and perceived responses of their colleagues and superiors to a PSI that occurred at their workplace in the past.

## Methods

### Study Design

To gain insight into medical professionals’ individual experiences and perceptions following PSIs and into their perceived level of psychological safety, we chose qualitative design of the research and method of individual semi-structured interview. Firstly, we prepared a research schedule for the interview, and then performed a pilot interview to test the intelligibility of the interview questions. One medical professional participated in a pilot interview. Interview schedule proved to be reliable, thus possible for use in research. Subsequently, from November 2022 to January 2023, we conducted individual interviews with medical professionals. Obtained data were recorded with the consent of respondents, transcribed verbatim and analysed by using conventional content analysis method and consensual qualitative research method.

### Sampling and Participants

Respondent selection was carried out using purposive and snowball sampling techniques. The main criterion for selection included the respondent working as a medical professional or that they had recent working experience at a healthcare facility at the time of conducting research. We selected predominantly medical professionals working in clinical workplaces exposed to a higher risk of PSI occurrence (surgery, oncology, etc.). Additionally, we ensured that our sample has an approximately equal representation of men and women and included were also participants with diverse lengths of clinical practice. After an interview, each respondent was asked to provide contact information on colleagues who could be approached to participate in the research.

### Procedure and Measures

Prior to the interview, each respondent received informed consent which specified the purpose of the research, terms of participation and the areas which the interview will be focused on. Respondents were assured that research is anonymous and voluntary. If respondents agreed to participate, they signed the informed consent. Interviews were conducted by the main author of this study. Individual interviews lasted approximately between 14 and 64 min and took place in person in various settings, mostly at a medical professional’s own workplace (at a specific healthcare facility) or in university settings.

During the interview, we asked respondents to provide socio-demographic information such as gender, age, highest educational level, current or last work position and length of clinical practice. Research schedule contained questions regarding medical professionals’ responses after an occurrence of a PSI: 1) respondents’ own responses or perceived responses of their colleagues (e.g., “How do you or your colleagues react if you witness that your colleague or superior is ignoring important safety rules, which could result in endangering patient safety?”), 2) perceived superiors’ responses (e.g., “If there was a PSI at your workplace, how did you perceive the response of your superiors to the given event?”).

### Data Analysis

In line with the qualitative design of the research, analysis was carried out using conventional content analysis method and consensual qualitative research method. Conventional content analysis is used in study designs focused on describing a complex phenomenon by gaining knowledge directly from participants’ perspectives [23], therefore it appeared to be a suitable approach for describing medical professionals’ individual responses and perceptions after a PSI. We also used elements of consensual qualitative research method, which included independent dual coding of the collected data by two coders and finding a common consensus about the meaning of the data between the coders [24]. Firstly, we performed the transcription of the data and uploaded the data into the MAXQDA software, version 2022. We familiarised ourselves with the data in order to enable the start of the analysis.

We created codes for each meaningful part of all the transcripts that captured the essence of the text. The data were independently dual coded by two members of the research team (LK and IS) in order to reach greater accuracy of the data. During the analysis later on, we consensually clustered similar codes into sub-categories and main categories. We repeatedly checked the consistency of sub-categories, as it was important that the sub-categories accurately describe codes assigned to them. The final list of categories and sub-categories was created based on a consensus of two research members (LK and IS). We also used diagram software to create a thematic map to depict the final categories and sub-categories of the data.

## Results

Research sample consisted of 16 medical professionals from Slovakia (62.5% females), specifically 12 physicians, 1 head of the unit, 1 head nurse, 1 nurse and 1 radiology technician. The average age of respondents was 38 years (SD = 11.74). The respondents’ length of clinical practice ranged between 4 months and 40 years. All respondents had completed higher level of education. Specifically, half of the respondents had completed the third degree of higher education (PhD), eight respondents have completed the second degree of higher education (master, MD) and 1 respondent had completed the first degree of higher education (bachelor). Socio-demographic characteristics of the sample are shown in Table 1.

**TABLE 1:** Socio-demographic characteristics of the sample (Psychological Safety in Healthcare study, Bratislava, Slovakia, 2024).

Characteristics	N	%
Age (mean/SD)	38.38	11.74
Length of clinical practice (mean/SD)	14.22	12.20
Gender		
Male	6	37.50
Female	10	62.50
Highest educational level		
The first degree of higher education (bachelor)	1	6.25
The second degree of higher education (master, MD)	7	43.75
The third degree of higher education (PhD)	8	50.00
Work position		
Physician	12	75.00
Senior physician	1	6.25
Head nurse	1	6.25
Nurse	1	6.25
Radiology technician	1	6.25

Across the data, we differentiated two categories of medical professionals’ responses to a PSI: 1) medical professionals’ own responses and their colleagues’ responses; and 2) superiors’ responses.

### Medical Professionals’ Own Responses and Their Colleagues’ Responses to a PSI

**FIGURE 1:**
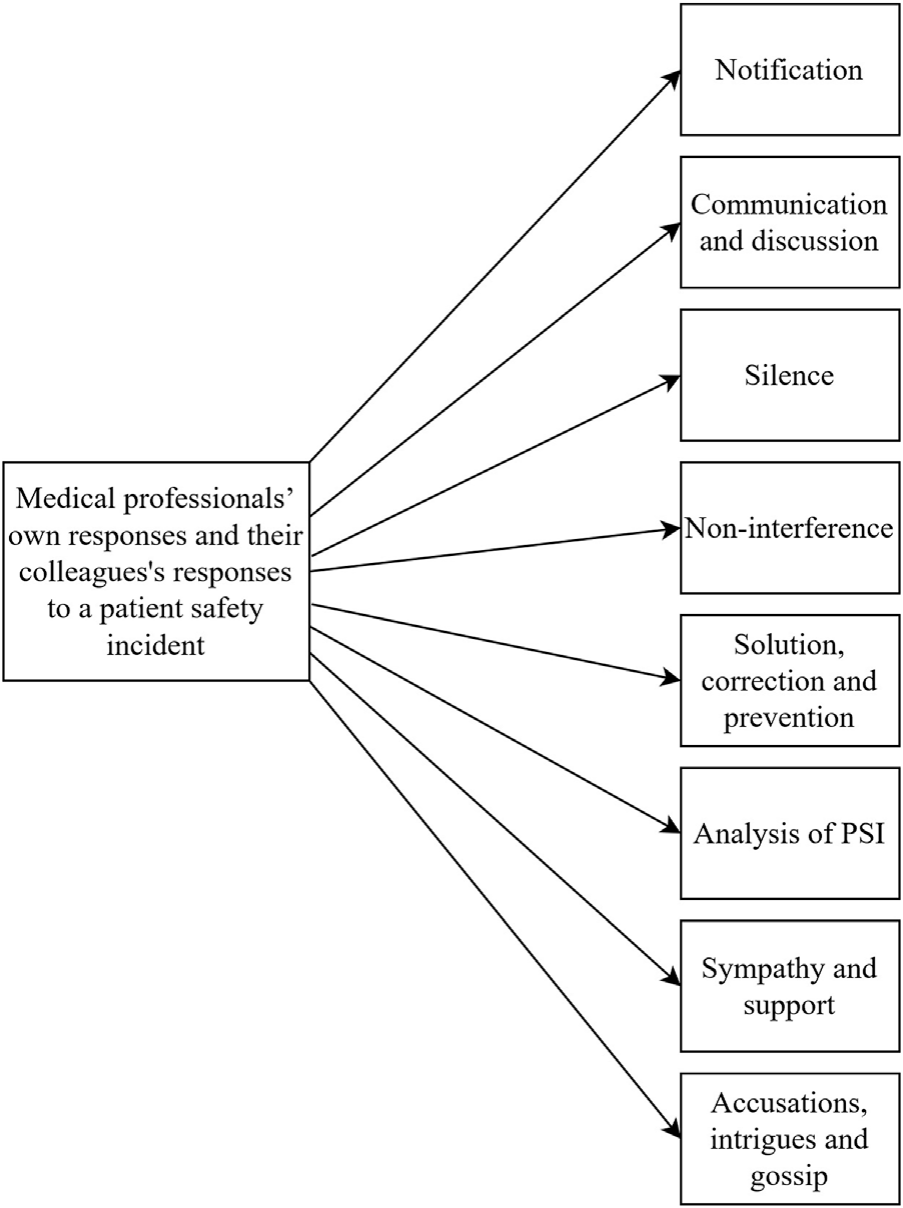
Diagram depicting the identified sub-categories of medical professionals’ own responses and their colleagues’ responses to a patient safety incident (Psychological Safety in Healthcare study, Bratislava, Slovakia, 2024).

Medical professionals respond in a variety of ways when they come across a PSI in their workplace. Some responses relate to the PSI itself, others relate to a specific medical professional involved in the PSI (either they were responsible for the occurrence of the PSI or were present when the PSI occurred). Eight sub-categories were identified (Figure 1): a) notification, b) communication and discussion, c) silence, d) non-interference; e) solution, correction and prevention, f) analysis of PSI, g) sympathy and support, h) accusations, intrigues and gossip. Table 2 shows the quotations representing each sub-category.

**TABLE 2:** Medical professionals’ own responses and their colleagues’ responses to a patient safety incident and illustrative quotations (Psychological Safety in Healthcare study, Bratislava, Slovakia, 2024).

Sub-categories	Quotations
Notification	“… in any case we will point out, we will point out the danger …”
Communication and discussion	“… I really try, if something like that happens, whether with a colleague or a superior, uh, I try to have a private conversation.”
Non-interference	“Someone would say that he/she didn't see it, he/she will not interfere.”
Silence	“… we have people in the team who keep silent, cover up, pretend that nothing is happening.”
Solution, correction and prevention	“… there is really an effort, whether it’s to prevent some worse complications or to arrange a remedy, to correct what has already happened.”
Analysis of PSI	“The given situation is discussed, it is said what could have been done differently or could potentially lead to a different result, … there’s a lot of talk about it and talk about whether something could have changed with the treatment or our intervention …”
Sympathy and support	“… we cry, we talk, we say to each other that ‘why’ (this has to happen), and we also deal with it over and over again, dissecting how it could happen …” “And it’s actually, uhm, very good that the older colleagues calm down the younger, less experienced one, and say that ‘it’s not as terrible as it looks, you have to do this, this and that, and it will simply be fine’, so …”
Accusations, intrigues and gossip	“There are colleagues who promptly point the finger (at someone), ‘it was him/her’, and it may not even be true …”

When medical professionals become aware of the risk of a PSI, they verbally *notify* someone about their patient safety concerns, whether in a direct or an indirect way. As an indirect notification, respondents mentioned expressing concerns in the form of a proposal or in the form of a naive question. The notification is followed by an open *communication and discussion* about the PSI. Respondents described they had private discussions with involved colleagues or superiors as well as joint discussions. However, medical professionals reported they have colleagues that do not talk about the PSI they have seen, or that they have personally caused it, they tried to cover it up (*silence*), or chose *not to interfere* in the PSI.

After a PSI happened, medical professionals reported that they try to do their best to find *a solution* and a way to *correct* what has been done for the patient’s benefit. Moreover, medical professionals talked about employing a *prevention* of similar situations in the future. *Analysis of PSI* as a standard practice to prevent PSIs in the future also occurred in the respondents’ statements.

According to the respondents, medical professionals who were responsible for a PSI—or were involved in a situation when a PSI happened—encountered mostly two types of responses from their colleagues. They received *sympathy and support* which included verbal support such as reassurance and encouragement to continue working as a medical professional, or practical support in the form of getting advice, or both. However, respondents also experienced *accusations, intrigues* and *gossip* in their workplace after a PSI.

### Superiors’ Responses to a Patient Safety Incident

**FIGURE 2:**
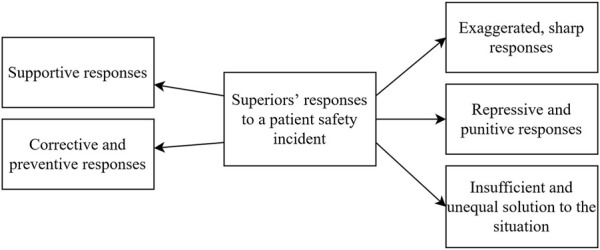
Diagram portraying the identified sub-categories of superiors’ responses to a patient safety incident (Psychological Safety in Healthcare study, Bratislava, Slovakia, 2024).

In our sample, medical professionals described superiors’ constructive responses as well as negative responses after a PSI had occurred in their workplace. Five sub-categories were identified (Figure 2): a) exaggerated, sharp responses; b) repressive and punitive responses; c) insufficient and unequal solution to the situation; d) supportive responses; e) corrective and preventative responses. Table 3 presents quotations representing each sub-category.

**TABLE 3:** Superiors’ responses to a patient safety incident and illustrative quotations (Psychological Safety in Healthcare study, Bratislava, Slovakia, 2024).

Sub-categories	Quotations
Exaggerated, sharp responses	“… it was very offensive, yes, (…) of course, things like that shouldn’t happen, but on the other hand, maybe you would expect a little support. … but it was pushed to such extreme conditions that (…) I don’t think these were adequate responses, downright implying that I can leave when I don’t like it here, when I can’t do it and so on …”
Repressive and punitive responses	“… the boss is trying to find the one to blame and make him feel it at least (…) That he is trying to find that one and draw attention to him in front of everyone.”
Superiors’ insufficient and unequal solution to the situation	“Uhm, some equality or, uh, I would say some absolutely fair approach, that whether it’s person A or person B, they, uh, communicate the same way or act the same way, that doesn’t, unfortunately, doesn't work like that here.”
Supportive responses	“… there are superiors, who are partners, who will support you, who will appreciate, who will appreciate the very thing that others do not even see. For example, they’ll appreciate that, uh … ‘It’s great that you were there at that moment, because it could have happened (something) worse.’”
Corrective and preventative responses	(about the superior) “He, in turn, took it into his own hands from such a structural matter and changed the procedure that led to what happened. He simply changed those recommendations to avoid it …”

Medical professionals described *exaggerated, sharp superiors’ responses* which included being reprimanded or being criticised. Respondents felt that these responses were inappropriate and unjustified given the situation and that they would expect a more supportive response, as the involved medical professional did everything in their power. Respondents had also experienced *repressive and punitive responses* in a sense that the superior was trying to find and punish the individual who they perceived as guilty. Respondents mentioned a few kinds of punishments–for example, the superior temporarily transferred the responsible person to another workplace or the superior actively pointed out the responsible one in front of others which led to a damaged reputation of that person. *Superiors’ insufficient and unequal solution to the situation* included the perceived insufficient drawing of consequences for the individual who was responsible for the PSI and an unequal approach from superiors regarding dealing with PSIs.

On the contrary, medical professionals described *supportive responses* from their superiors. This included getting verbal support (e.g., reassurance that PSIs happen to every medical professional or getting recognition for handling the PSI) or receiving practical support in the form of finding the right solution to the PSI. Medical professionals also acknowledged that when a PSI happened, their superiors made every effort to redress the situation and take action to prevent a similar situation from happening again (*corrective and preventive responses*).

## Discussion

In the present study, we explored psychological safety manifested in medical professionals’ responses to PSIs occurring in Slovak healthcare facilities.

Our results captured the medical professionals’ efforts to act for the benefit of patients by openly communicating about the PSI with other people or by taking steps to resolve and prevent the PSI. However, passive responses to a PSI which threaten patient safety were captured as well. Research studies show that perceived risk of patient harm is often the motivation for healthcare workers to speak up or report a PSI [19, 25]. Notification as one of our identified responses to a PSI is similar to what Tarrant et al. (2017) described as “pre-emptions” in their study—a safe way to point out risky behaviour and prevent patient harm [26]. Therefore, open or assertive communication leads to better patient safety outcomes [13, 14, 27]. Despite the evident motivation to help the patient, episodes of silence after a PSI occurred as a sub-category in our study, most likely due to fear of expected negative consequences [25, 28]—e.g., fear of being blamed [28], fear of punitive measures [29], or fear of conflicts [19]. Occurrence of silence after a PSI could imply insufficient psychological safety, as a high level of perceived psychological safety reduces the tendency to remain silent [30]. Our results also show that the occurrence of a PSI requires an immediate corrective action by medical professionals followed by analysis of PSI. Previous study indicated that root-cause analysis of PSIs seems to receive insufficient attention from the hospital management [7]. However, it is the analysis that is crucial for eliminating the systemic causes of PSIs and improving patient safety [31]. Following a PSI, medical professionals involved in the incident tend to seek support from people they trust [32], and as our results show, they mainly turn to colleagues for help. Receiving immediate support will allow the medical professionals to effectively cope with a PSI [33]. On the contrary, we discovered that medical professionals experienced accusations or gossip from their colleagues, which is in line with a recent Slovak study concluding that experiencing a PSI is related to conflicts among colleagues [7]. Non-supportive responses from coworkers could ultimately have a negative impact on responsible medical professionals—for example, in the form of experiencing self-doubt or loss of clinical confidence [32].

Medical professionals in our sample experienced or witnessed both positive and negative responses from superiors after a PSI, which is also reflected in previous research studies [22, 26, 33–35]. Regarding negative responses, medical professionals responsible for a PSI or involved in a PSI experience unfair treatment [34, 35], being blamed [22, 34, 35], punished [22, 26], scolded [26] or denounced by their superiors [22, 26]. Experiencing or merely witnessing superiors’ negative responses after a PSI could reinforce fear to speak up about PSIs [14, 17, 21], thus seem to be detrimental to the perception of psychological safety in the workplace [13]. Nevertheless, medical professionals in our sample received emotional or practical support from superiors after a PSI, and these types of support align with findings from previous research studies [22, 33, 34]. Additionally, our study depicted preventive measures taken by superiors to prevent a PSI in the future, allowing change in clinical practice and learning from a PSI [8, 31]. Both positive and negative responses to a PSI occurred in our interviews. Therefore, it is important to note that inability to predict the superior’s response to speaking up—uncertainty whether they will receive support or a negative response—may lead to episodes of silence as well [25].

### Strength and Limitations

Present study has several strengths. The use of individual interviews allowed us to explore Slovak medical professionals’ experiences with PSIs during their clinical practice, while shedding light on some similarities and differences in medical professionals’ behaviours towards a colleague in comparison to a superior. Moreover, we contributed to the current knowledge by outlining a connection between medical professionals’ responses after a PSI and a climate of psychological safety in the workplace.

Limitations of the study should be carefully considered, as well. The small sample size is the primary limitation of the study, despite the fact that we achieved sufficient saturation of categories and sub-categories of the data. Secondly, the descriptive nature of the study limited the interpretative power of the results. It is also important to remember that PSIs are a sensitive issue, which might initiate social desirability and ultimately result in censored information provided by respondents. However, we tried to create a safe environment during the interviews and assured the respondents about the anonymity of the research in order to reduce these tendencies. Last but not least, despite the researchers’ efforts, our research sample was not diverse in terms of the hierarchical position of medical professionals. Therefore, more research studies focusing on perspectives of nurses, head nurses, senior physicians and other medical professionals are needed.

### Implications for Practice and Future Research

In terms of implications for practice, results of this study pointed to the poor interpersonal communication mostly between superiors and medical professionals involved in a PSI. Therefore, the focus should be on moving beyond a culture of blame and promoting open and supportive communication [36]. In order to foster an atmosphere of psychological safety, it is important that superiors normalise PSIs in healthcare and frame them as learning opportunities for medical professionals rather than viewing them as an act of individual failure [17]. It is equally important that retrospective analysis of PSIs would be a standard practice in the clinical departments. Using a systems approach to the analysis of PSIs demonstrated in the study by Leveson et al. would also prevent negative responses to medical professionals involved in a PSI [31]. Future research could focus on exploring responses to PSIs and psychological safety in specific groups of medical professionals (among nurses, physicians, etc.) to gain insight into the differences between these groups. This could help to design future interventions aiming to enhance psychological safety and open communication about PSIs better tailored to needs of specific groups of medical professionals.

### Conclusion

Medical professionals’ responses to PSIs occurring in healthcare facilities are diverse, which implies the potential for fostering and enhancing the climate of psychological safety, so that all medical professionals feel safe to openly communicate about PSIs with their coworkers regardless of their position in the medical hierarchy.
